# *Zataria multiflora* could improve hippocampal tau protein and TNF_α_ levels and cognitive behavior defects in a rat model of Alzheimer's disease 

**Published:** 2019

**Authors:** Mohabbat Ahmadi, Mahnaz Taherianfard, Tahora Shomali

**Affiliations:** 1 *Physiology division of Basic Science Department, School of Vet Med; Shiraz University, Shiraz, Iran.*; 2 *Pharmacology division of Basic Science Department, School of Vet Med; Shiraz University, Shiraz, Iran.*

**Keywords:** Alzheimer’s disease, Zataria multiflora, Morris water maze, Tau protein, TNFα

## Abstract

**Objective:**

*Zataria multiflora* (ZM) is a plant with ethnopharmacological value which was recently tested to reduce symptoms of Alzheimer’s disease (AD). The aim of the present study was to determine the effect of ZM essential oil on spatial cognitive and noncognitive behavior, as well as hippocampal tau protein and tumor necrosis factor alpha (TNFα) concentrations in rats with AD.

**Materials and Methods:**

Thirty-five adult male Sprague Dawley rats (300±30 g) were randomly divided into 5 groups: control (intact rats); sham (received intracerebroventricular (ICV) microinjection of normal saline); AD control (rats with AD that did not receive any treatment); vehicle control (rats with AD that orally received tween-80, 5% (ZM essential oil vehicle) for 20 days) and experimental (rats with AD that orally received ZM essential oil 100 µl/kg/day for 20 days). AD was induced by bidirectional microinjection of β amyloid 1-42 (10 µg/2µl). Tau protein and TNFα concentrations were measured by ELISA methods. Spatial cognitive and noncognitive behavior were determined by Morris water maze (MWM) test.

**Results:**

ZM essential oil significantly improved latency time, time spent in the target quarter and cognitive behavior of rats with AD compared to control and sham groups (p<0.05). Hippocampal tau protein and TNFα concentrations were significantly higher in both AD control and vehicle groups compared to control and sham groups respectively (p<0.01 and p<0.001), administration of ZM essential oil reduced these parameters as compared to AD control and vehicle groups respectively (p<0.01 and p<0.001).

**Conclusion:**

ZM essential oil improves spatial learning and memory of rats with AD as assessed by MWM test. These effects are associated with decreased concentrations of hippocampal tau protein and TNFα.

## Introduction

Alzheimer's disease (AD) is a progressive neurodegenerative disorder. It is associated with memory and cognition impairment and may finally lead to death (Davinelli et al., 2012 ▶). This disease is the most common reason of dementia in old people with increasing prevalence due to global increase in population life expectancy (Adlard et al., 2014 ▶). 

Neuropathological features of AD are classically the presence of amyloid plaques and intracellular neurofibrillary tangles of hyper-phosphorylated tau protein, accompanied by inflammatory changes (Gazova et al., 2012 ▶). Aging plaques are formed by the accumulation of β amyloid peptides, consisted of 40 or 42 amino acids (Aβ40 and Aβ42, respectively). Plaques are observed in the brain structures, such as the hippocampus and the entorhinal cortex. Accumulation of high levels of β amyloid peptides leads to apoptosis associated with AD (Wirths et al., 2004 ▶). β amyloid triggers astrocytes and microglia to release cytokines and inflammatory factors including tumor necrosis factor alpha (TNFα) which has a proven role in a number of neurodegenerative disorders such as AD (Rodney et al., 2018 ▶).

Attachment of β amyloid to microglial cell surface promotes expression of pro-inflammatory cytokines such as TNFα which leads to tau hyper-phosphorylation and subsequent neuronal loss (von Bernhardi et al., 2010 ▶). Tau protein is a signaling molecule (Arendt et al., 2016 ▶) and its phosphorylation results in its detachment from microtubules and production of higher levels of soluble free tau protein. Finally, tau protein hyper-phosphorylation leads to neurofibrillary tangles (NFTs) production and neurotoxicity which are indicators of AD (Keck et al., 2003 ▶; Ren et al., 2007 ▶). NFT-induced reduction in proteasome activity results in an abnormal accumulation of proteins and initiates a cascade of events which finally leads to neuronal death (Ying et al., 2012 ▶).

In addition to conventional drug therapy for management of cognitive disorders, herbs have been traditionally used to enhance cognition and improve AD symptoms (May et al., 2009 ▶). *Zataria multiflora* (ZM) is a member of the Lamiaceae family which is native to Iran, Afghanistan and Pakistan (Ali et al., 2000 ▶). Its main constituents are thymol, stigmasterol, p-cymene, linalool, flavonoids and carvacrol (Sajed et al., 2013 ▶). Thymol, linalool and carvacrol have shown anticholinesterase properties (Jukic et al., 2007 ▶) which are beneficial to AD management. Essential oil and methanolic extract of ZM also exerted anticholinesterase activities (Sharififar et al., 2012). The essential oil of this plant ameliorates AD symptoms in laboratory models as shown by Morris water maze (MWM) test (Majlessi et al., 2012 ▶; Eskandari-Roozbahani et al., 2019 ▶) which is a simple procedure to determine the spatial cognitive and noncognitive behavior (Vorhees and Williams, 2006 ▶). Noncognitive behavior of MWM is characterized by latency time and time spent in target quarter. These factors are simply quantified and give an incomplete interpretation of MWM paradigm while the cognitive behavior of MWM provides complementary data. Cognitive behavior is characterized by swimming strategies such as direct, corrected, circling, and thigmotaxis patterns*. *In 2016, Illouz et al. presented the MWM unbiased strategy classification (MUST-C) algorithm, which provides a standardized method of strategy classification as well as a cognitive scoring scale. In this algorithm, cognitive performance of each training trial is scored according to cognitive strategies. Thigmotaxis sore is 1 in this algorithm and is the specific characteristic of AD (Illouz et al., 2016 ▶). 

Knowledge is scarce on ZM essential oil and AD and their relation to TNFα, and we did not find any research on the effect of ZM essential oil on hippocampal tau protein and cognitive behavior. Therefore, the present study investigated the effect of ZM essential oil on latency time to reach to the platform, time spent in the target quarter and the percentage of thigmotaxis behavior (the number of thigmotaxis behavior/4×100) as a measure of anxiety and searching strategy in MWM spatial learning and memory test as well as hippocampal tau protein and TNF_α_ concentrations in male rats with experimental AD. 

## Materials and Methods

**Materials**

Tween-80 (CAS Number: 9005-65-6), Aβ1-42 peptide (AG968) and Congo red solution were purchased from sigma-Aldrich Co. ZM pure essential oils were purchased from Barij essence pharmaceutical company, Iran (voucher Number: 41754). Ketamine and xylazine were purchased from Alfasan, Netherlands. Tau protein (Rat phospho tau protein ELISA kit, Cat. No. E1388Ra) and TNFα (Rat tumor necrosis factor α ELISA kit, Cat. No. E0764Ra) were purchased from Bioassay Technology Laboratory, China.

**Animals**

In this study, 35 male Sprague Dawley rats weighing 300±30 g were used and kept under standard conditions of temperature 22±2, humidity 35 to 45% and the lighting conditions of 12 hours of light and 12 hours of dark. Animals had access to food and water *ad libitum* and they were randomly divided into 5 groups (n=7) as follows: 1- Control group: intact rats; 2- Sham group: received 2 µl of normal saline bilaterally at 0.5 μL/min in the lateral ventricles of the brain on each side. 3- AD control group: rats received 10 μg/2μl of Aβ1-42 peptide by stereotaxic surgery at a rate of 0.5 μL/min for induction of AD (Majlessi et al., 2012). 4- Vehicle control group: rats received 10 μg/2μl of Aβ1-42 peptide by stereotaxic surgery at a rate of 0.5 μL/min for induction of AD + tween-80 5% (as ZM essential oil vehicle) for 20 days by oral gavage. 5- Experimental group: rats received 10 μg/2μl of Aβ1-42 peptide by stereotaxic surgery at a rate of 0.5 μL/min for induction of AD + ZM essential oil 100 μl/kg/day in tween-80 5% by oral gavage (Majlessi et al., 2012) for 20 days.

In all groups, spatial learning and memory were assayed from day 21 by MWM test for one week.

**AD induction**

Animals were anesthetized using intra peritoneal (IP) injection of ketamine 10% (100 mg/kg) and xylazine 2% (10 mg/kg). Rats were fixed in the stereotaxic apparatus and Aβ1-42 peptide was injected bilaterally into lateral ventricle according to Paxino and Watson atlas, 0.5 mm posterior to Bregma; 1.5 mm lateral to midline and 3.5 mm above the lateral ventricle (Majlessi et al., 2012 ▶). 

Morris water maze (MWM) test: 

MWM is made of a circular water tank (diameter: 110 cm, and height: 60 cm) which has a black inner surface. Inside the tank, there is a small removable platform with surface area of 10 cm^2^, black and unpolished. Circular reservoir is divided into four-directional parts namely, northeast (NE), northwest (NW), southeast (SE), and southwest (SW) and added cue symbols to all of them. These cues were geometric shapes with black lines and were hung from the inner wall of the tank. The MWM test was performed in a sound-insulated room with light intensity of 20 lux. A digital camera placed above the pool level, captured rat's movements at any moment and transferred them to a computer equipped with NeuroVision software made by Tajhis Gostar Co. 

**Behavioral testing**

MWM was performed after twenty days of administering ZM essential oil and continued for one week. The procedure was done as follows. During the first three days, training was performed with the visible platform to familiarize animals with MWM. In each training period, the animals were put in the pool in a way that rats were facing the wall of the pool. The location to start the animal abandonment in the pool was determined randomly (SW, SE, NE, or NW). The rats were then allowed to find a rescue platform during 90 sec of swimming in the pool. If the animal was not able to find the platform during this period, it would be guided by the experimenter to the platform. After the animal arrived at the platform, it was allowed to stay for 30 sec on the platform to recognize the platform position. Then, it was removed from the pool and after 30 sec of rest in the cage, the experiment was repeated from another quarter. This process was repeated in four directions, and each rat was trained four trials daily in various quarters to reach the apparent platform every day. During the second three-days, the procedure was performed in a completely similar manner to that of the first three days but with a hidden platform (1 cm below the water level). On the seventh day (i.e. the probe test day), the platform was removed and the animals were allowed to swim in the tank for 90 sec. Various parameters were determined according to the hypothetical platform location in the NeuroVision software and the animal's passage through hypothetical platform. The parameters determined in the present study were as follows. The delay time required for reaching the platform and the time spent in the target quarter as noncognitive behaviors and the percentage of thigmotaxis behavior as cognitive behavior. For determination of the percentage of thigmotaxis, the number of thigmotaxis behavior was compared to other behaviors, so that in 4 trails, if a rat exhibited a single thigmotaxis behavior, the percentage was considered 25%, and statistical analysis was performed according to above percentage; then, during all tests the platform remained in the fixed quarter. In the statistical analysis, the data of platform quadrant were considered (Illouz et al., 2016 ▶; Mendez et al., 2008 ▶).

**Brain tissue preparation**

Immediately after euthanizing the animals according to animal welfare guidelines, the whole brain was removed. The right hippocampus was immediately transferred to a -80C freezer for later examination. To measure the factors, the samples were first homogenized in 0.1 M phosphate buffer solution (pH 7.4), and then after centrifugation, supernatant fluid was used to measure the tau protein and TNFα concentrations. The left hippocampal tissue was fixed in formalin 10% for histopathological examinations (Congo red staining). 

**Histopathological examination (Congo red staining)**

The method introduced by Wilcox et al., was used to detect amyloid plaques in the hippocampus for confirmation of AD induction (Wilcock et al., 2006 ▶). Then, microphotographs were taken from the slides of different groups by a camera attached to microscope.

**Statistical analysis**

Data were analyzed using SPSS software (Version 21). One-Way ANOVA and Tukey's test as *post-hoc* were used to analyze all parameters. Results are reported as mean±SEM. The significance level was set at p<0.05.

All procedures involving animal subjects were reviewed and approved by the Institutional Research Ethics Committee of the School of Veterinary Medicine, Shiraz University (approval No.:96INT1M1755).

## Results

In the present study, MWM test and Congo red staining were used for the following 2 purposes: 1- confirmation of AD induction, and 2- To detect the possible effect of ZM on AD.

**Figure 1:**
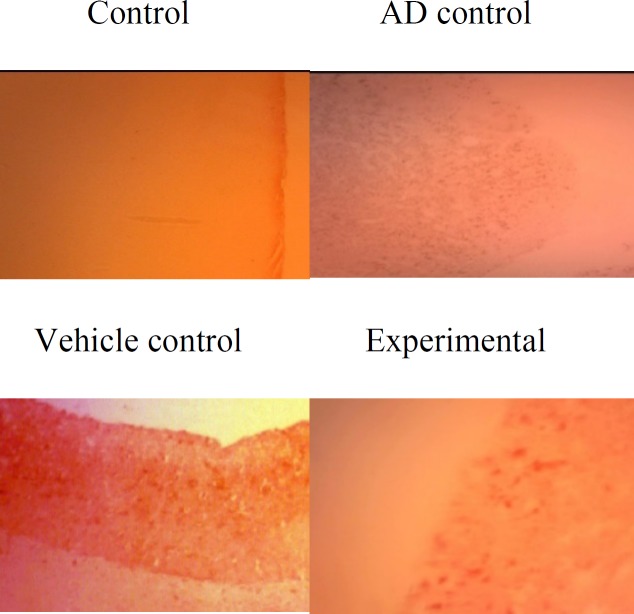
Photomicrographs presenting the effect of ZM essential oil on β amyloid plaques in hippocampus of rats with β1-42 amyloid-induced AD (magnification: 40×10)

Congo red staining clearly showed plaque formation in the hippocampus of rats with AD. In rats treated with ZM essential oil, density of β amyloid plaques was reduced (Figure 1). 

ZM=* Zataria multiflora*; AD= Alzheimer’s disease.

**Figure 2:**
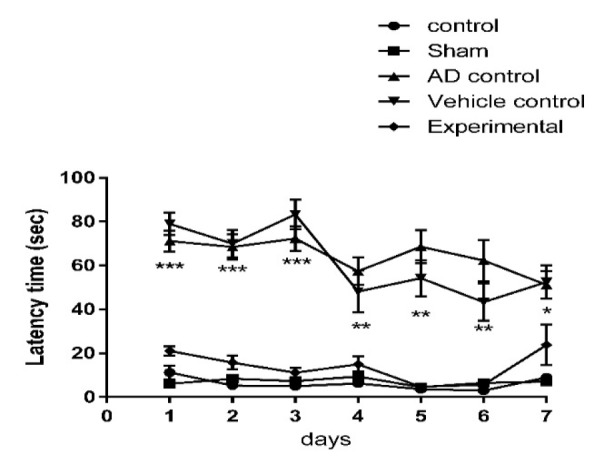
The effect of ZM essential oil on latency time to reach platform in MWM test during 7 days of test (3 days of appearance platform, 3 days of disappearance platform and one day without platform) in rats with AD induced by β1-42 amyloid. * p<0.05 for both AD and vehicle control compared to other groups; ** p<0.001 for both AD and vehicle control compared to other groups; and *** p<0.0001 for both AD and vehicle compared to other groups. ZM*= Zataria multiflora*; MWM= Morris water maze; AD= Alzheimer’s disease

**Figure 3:**
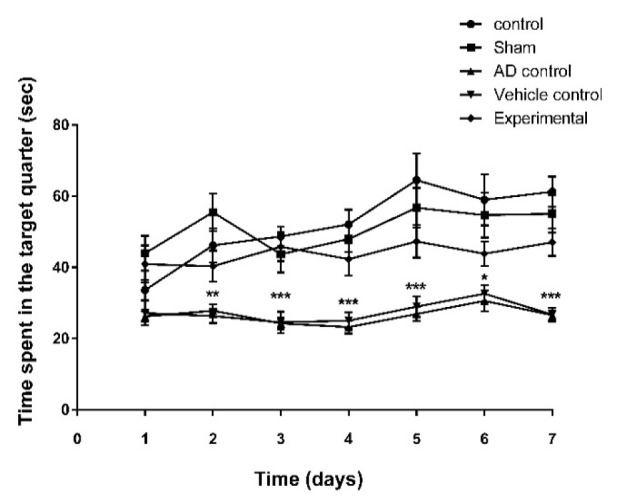
The effect of ZM essential oil on the time spent on target quarter in MWM test during 7 days of test (3 days of appearance platform, 3 days of disappearance platform and one day without platform) of AD induction by β1-42 amyloid in rats. * p<0.05 for both AD and vehicle control compared to other groups; ** p<0.001 for both AD and vehicle control compared to other groups; and *** p<0.0001 for both AD and vehicle control compared to other groups. ZM= *Zataria multiflora*; MWM= Morris water maze; AD= Alzheimer’s disease

 Data from MWM test showed that latency time to reach the platform significantly (p=0) increased in AD and vehicle controls compared to other groups (Figure 2). There was no significant difference among experimental, control and sham groups. In probe test (without platform) the time spent in target quarter in both AD and vehicle control groups was significantly shorter (p<0.0001) than other groups. The experimental group also showed significantly (p<0.05) shorter time than control and sham groups; however, this time was significantly (p<0.0001) longer in experimental group than both AD and vehicle control groups (Figure 3).

**Figure 4:**
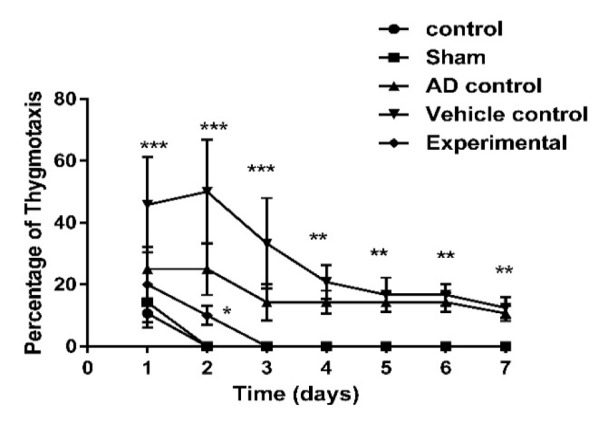
The effect of ZM essential oil on percentage of thigmotaxis behavior in MWM test during 7 days of test (3 days of appearance platform, 3 days of disappearance platform and one day without platform) of AD induction by β1-42 amyloid in rats. * p<0.05 for both AD and vehicle control compared to other groups; ** p<0.001 for both AD and vehicle control compared to other groups; and *** p<0.0001 for both AD and vehicle control compared to other groups. ZM= Zataria multiflora; MWM= Morris water maze; AD= Alzheimer’s disease

On the first day, thigmotaxis behavior was observed in all groups, but on the second day, it reduced to zero percent in control and sham groups. On the third day, this behavior was not observed in the experimental group. However, in both AD and vehicle controls, this behavior was present even on the seventh day of the experiment (Figure 4).

**Figure 5:**
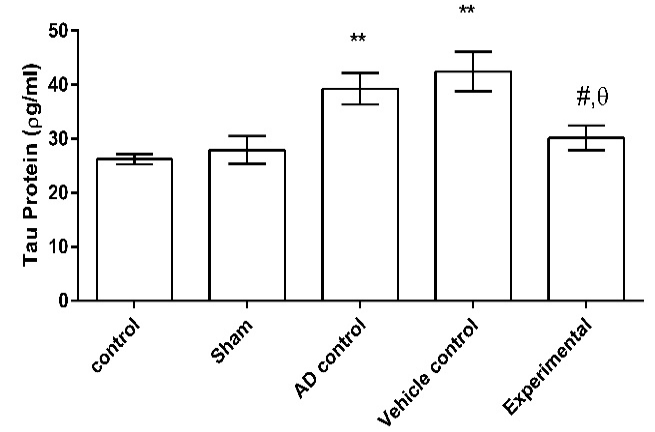
The effect of ZM essential oil on tau protein level in hippocampus of rats with AD induced by β1-42 amyloid. ** p<0.01 compared to control and sham groups; # p<0.01 compared to AD control; and θ p<0.0001 compared to vehicle control. ZM= *Zataria multiflora*; AD= Alzheimer’s disease

Concentration of tau protein in hippocampus was significantly higher in both AD and vehicle controls as compared to control and sham groups (p<0.01) as well as the experimental group respectively (p<0.01 and p<0.001). The tau protein concentration was not significantly different among experimental, control and sham groups (p>0.05) (Figure 5).

**Figure 6:**
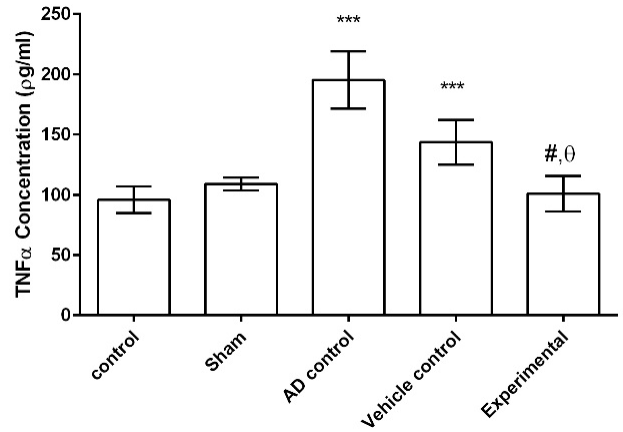
The effect of ZM essential oil on TNFα concentration in hippocampus of rats with AD induced by β1-42 amyloid. *** p<0.001 compared to control and sham groups; # p<0.01 compared to AD control; and θ p<0.0001 compared to vehicle control. ZM= *Zataria multiflora*; TNFα= tumor necrosis factor alpha; AD= Alzheimer’s disease

Figure 6 shows that the concentration of TNFα in hippocampus was significantly higher in both AD and vehicle controls as compared to control and sham groups (p<0.001 for all cases). There was no significant difference in TNFα concentration among experimental, control and sham groups (p>0.05). Concentration of TNF-α in hippocampus of experimental group was significantly lower than that of the AD control (p<0.01) and vehicle control (p<0.0001).

## Discussion

In this study, some of the possible mechanisms for the protective effect of ZM essential oil on AD were evaluated with respect to important factors that contribute to the induction and progression of this disorder. The Congo red staining and MWM test were used to confirm the induction of AD. A high density of β-amyloid plaques in the brain, increased time to reach the platform, reduced time spent in target quarter and the persistence of thigmotaxis behavior in the MWM test indicated that AD was successfully induced by Aβ1-42. On the other hand, reduced amyloid deposits in the hippocampus, decreased time to reach the platform, increased in time spent in target quarter and diminished thigmotaxis behavior of rats that were treated with ZM essential oil, demonstrated that ZM essential oil can reduce the noncognitive and cognitive behavioral disorders. In fact, pathological and behavioral changes were consistent. 

One of the symptoms of behavioral defects in AD is anxiety. Thigmotaxis is generally thought to be an indicator of anxiety (Treit and Fundytus, 1988 ▶). When thigmotaxis occurs, rodents can seldom find the platform since the exploration process is disturbed. Therefore, the frequency of this behavior is an important factor in assessment of an animal’s spatial learning ability. Some reports stated that anxiety or emotional stress can impair spatial learning and memory (Goodman and McIntyre, 2017 ▶). The present results indicate that in both AD and vehicle controls, thigmotaxis behavior existed even on the seventh day of the experiment that is consistent with previous studies as Petrasek et al. (2018) ▶ reported higher thigmotaxis in the McGill rats (an animal model of the familial AD) relative to control group (Petrasek et al., 2018 ▶). Moreover, in present study, thigmotaxis reached zero percent in experimental group. Therefore, we can say that consumption of ZM essential oil can be helpful for reducing the anxiety symptoms of AD. 

Based on the results of the present study, ZM essential oil reduced the level of tau protein, which is important in neural protection in the treatment of AD. Bakhtiari et al. (2017) ▶ reported the role of flavonoids (one of the ZM essential oil compounds) in inhibiting specific enzymes involved in tau protein phosphorylation, such as β-secretase, glycogen synthase and kinase-dependent cyclin 5, all of which could contribute to beneficial effects of flavonoids on AD (Bakhtiari et al., 2017 ▶). 

*P*-cymene is one the most important constituents of ZM essential oil. Recently, Haribabu et al. reported a decrease of 60-70% in the accumulation of β amyloid peptide by p-cymene complexes 1 and 2 (Haribabu et al., 2017 ▶). Also, it was shown that cognitive impairment caused by an increase in β amyloid is affected by the anti-inflammatory, anti-oxidant, and anticholinergic properties of thymol (Azizi et al., 2012 ▶). Therefore, it can be expected that the ZM essential oil through its thymol content can mitigate the progression of AD. In addition, the sterol compounds of ZM essential oil may also be important in reducing AD complications. Burg et al (2013) ▶ reported that phytosterols such as stigmasterol can reduce the production of β amyloid by different mechanisms (Burg et al., 2013 ▶). 

Another finding of the present study was that oral administration of ZM essential oil reduces the amount of pro-inflammatory cytokine TNFα in hippocampus of rats with AD. Previous studies indicated that cell apoptosis in neurological degeneration is based on the production and secretion of pro-inflammatory mediators in diseases such as AD, and brain inflammation is a pathological hallmark of AD (Rubio-Perez and Morillas-Ruiz, 2012 ▶). Linalool (which is also present in ZM essential oil) can reduce the cognitive impairment induced by amyloid β, owing to its neuro-protective properties mediated via reducing apoptosis and oxidative stress and anti-inflammatory effects attributed to the blockade of MAPK (mitogen-activated protein kinases) and NF-B (nuclear factor kappa-light-chain-enhancer of activated B cells) activity (Gunaseelan et al., 2017 ▶).

ZM essential oil improves spatial learning and memory of rats with AD as shown by MWM test. These effects are associated with a decrease in hippocampal tau protein and TNFα concentrations.
